# Effects of vitamin D supplementation on the bone specific biomarkers in HIV infected individuals under treatment with efavirenz

**DOI:** 10.1186/1756-0500-5-204

**Published:** 2012-04-26

**Authors:** Maryam Etminani-Esfahani, Hossein Khalili, Sirous Jafari, Alireza Abdollahi, Simin Dashti-Khavidaki

**Affiliations:** 1 Clinical Pharmacist, Department of Clinical Pharmacy, Faculty of Pharmacy, Ahvaz University of Medical Sciences, Tehran, Iran; 2 Clinical Pharmacist, Department of Clinical Pharmacy, Faculty of Pharmacy, Tehran University of Medical Sciences, Tehran, Iran; 3 Iranian Research Center for HIV/AIDS, Department of Infectious Diseases, Faculty of Medicine, Tehran University of Medical Sciences, Tehran, Iran; 4 Department of pathology, Faculty of Medicine, Tehran University of Medical Sciences, Tehran, Iran; 5 Department of Clinical Pharmacy, Faculty of pharmacy, Tehran University of Medical Sciences, Enghelab Ave, 1417614411, P.O.Box:14155/6451, Tehran, Iran

**Keywords:** HIV, Efavirenz, Vitamin D

## Abstract

**Background:**

It was reported that antiretroviral drugs such as efavirenz can increase the catabolism of vitamin D in HIV infected individuals. We have not found any study that evaluated effects of vitamin D supplementation on the bone specific biomarkers in HIV positive patients under treatment with antiretroviral regimen containing efavirenz.

**Findings:**

Vitamin D deficiency was detected in 88.4 % of included patients. Baseline osteocalcin, but not collagen telopeptidase, serum levels were lower than normal range in all of these individuals. Both bone biomarkers’ concentrations increased significantly (p < 0.001 for both of them) after supplementation of vitamin D and it was more predominant for osteocalcin.

**Conclusion:**

In the HIV-infected patients under treatment with efavirenz, vitamin D deficiency is prevalent. After supplementation with single dose of 300,000 IU vitamin D in this population, the activation of osteoblasts and osteoclasts stimulates bone formation and resorption respectively with favorable bone formation without any adverse event. Significant percent of HIV infected individuals are vitamin d deficient that could benefit from vitamin D supplementation.

## Findings

### Background

Human immunodeciency virus (HIV) infection is a global health care problem and about 33 millions individuals living with this infection around the world [1]. Following screening program and early diagnosis of HIV infection, introducing of effective antiretroviral therapy and improvement of patient’ care; survival increased and consequently chronic diseases such as bone disorders diagnosed more frequently in this population [2]. Studies have shown that bone disorders are more prevalent in HIV positive patients in comparison with HIV negative individuals who were at the same age range, sex and race [3-8]. Associations between bone mineral density decline and duration of HIV infection, HIV viral load and CD4 cells count was reported [9].

HIV infected individuals are more vulnerable to micro-elements malnutrition due to decreased intake, mal-absorption, increased need, metabolism changes and antiretroviral therapy. Vitamin D deficiency, as a fundamental element for regulation of bone metabolism, was reported more frequently in this population [2]. Also it was reported that antiretroviral drugs such as efavirenz can increase the catabolism of vitamin D in HIV infected individuals [10]. Recently efavirenz was proposed as a risk factor for vitamin D deficiency in this population [10-12]. We have not found any interventional study that showed the effects of vitamin D supplementation on the bone specific biomarkers in HIV positive patients under treatment with antiretroviral regimen including efavirenz. In present study we have evaluated the effects of vitamin D supplementation on bone specific biomarkers including osteocalcin (OC); an osteoblast metabolic marker and collagen telopeptidase (CTx); a bone collagen degradation byproduct as indicator of bone resorption [7] in vitamin D deficient HIV positive individuals under treatment with antiretroviral drugs including efavirenz.

### Results

From 121 HIV positive individuals that had the study inclusion criteria, 107 of them had vitamin D deficiency (88.4 %) and included in intervention phase of the study. From included individuals, 98 of them (74 male and 24 female with mean ± SD ages of 40.25 ± 8.94 years old) completed the study. Nine patients excluded from the study due to follow-up problems. The median period past from the patients’ efavirenz starting date up to enter the study was 15.5 months with a range of 1–49 months. The patients’ demographic and clinical data have been shown in the Table 1.

**Table 1:** The demographic, baseline clinical characteristics of the patients

**Characteristic**	**Frequency, mean or median**
Sex, number (percent)	
Male	74(75.5)
Female	24(24.5)
Age, mean(SD), years	40.26 ± 8.99
Injection drug abuse, number (percent)	
Yes	55(56.1)
No	43(43.9)
Duration since HIV diagnosis, median (range), month	26.5 (3–152)
Chronic hepatitis B, number (percent)	
Yes	6(6.1)
No	92(93.9)
Chronic hepatitis C, number (percent)	
Yes	53(54.1)
No	45(45.9)
GFR, mL/min/1.73 m^2^, number (percent)	
≥90	75(76.6)
<90	23(23.4)
Calcium level mg/dl, mean (SD)	8.97 ± 0.40
Phosphorus level mg/dl, mean (SD)	3.25 ± 0.69
Parathyroid hormone level pg/ml, mean (SD)	33.92 ± 17.96
Alkaline phosphatase IU/l, mean (SD)	200.80 ± 72.05
Antiretroviral regimen, number (percent)	
Standard (Zidovudine + lamivudine + Efavirenz)	88(89.7)
Other	10(10.3)

GFR: Glomerular filtration rate.

The patients’ vitamin D and bone biomarkers’ levels at baseline and after three months of vitamin D administration were shown in the Table 2. Baseline OC levels were lower than normal range (5–25 ng/ml) in all of the HIV infected individuals whereas CTx levels were in the normal range (0.11-0.75 ng/ml) for included patients. Both bone biomarkers’ concentrations increased significantly after supplementation of vitamin D but these changes was not equal. OC concentration raised 208 percent while CTx increased about 60 percent above baseline values.

**Table 2:** Serum levels of vitamin D and bone biomarkers before and after vitamin D supplementation

**Parameter**	**Baseline (pre-intervention)**	**Three months later (post-intervention)**	***P* value**
Serum 25-OH vitamin D (nmol/L)	18.54 ± 12.92	96.10 ± 37.05	<0.001
PTH (pg/ml)	33.92 ± 17.96	22.53 ± 18.69	<0.001
Serum ALP (IU/L)	200.80 ± 72.05	273.52 ± 96.55	<0.001
Serum Osteocalcin (ng/ml)	3.60 ± 3.76	11.09 ± 7.32	<0.001^*^
Serum CTx (ng/ml)	0.48 ± 0.25	0.77 ± 0.40	<0.001

ALP: Alkaline Phosphatase; CTx:Collagen Type 1 C-Telopeptide; PTH: Parathyroid Hormone.

* Sign test was used to evaluate the difference between pre and post intervention values.

Serum OC levels had positive correlations with vitamin D serum level (r = 0.38, *p* < 0.001), serum alkaline phosphatase (r = 0.38, *p* < 0.001), CTx concentration (r = 0.47, *p* < 0.001) and serum Phosphor level (ALK) (r = 0.25, *p* = 0.013). Also OC serum level was significantly lower in HIV/HCV (hepatitis C virus) co-infected patients than HCV negative individuals (*p* = 0.03).

Patients’ serum levels of CTx had statistically significant inverse correlations with glomerular filtration rate (GFR) (r = −.025, *p* = 0.02). Conversely, CTx levels had positive associations with serum levels of ALP (r = 0.35, *p* < 0.001), serum P concentration (r = 0.21, *p* = 0.04), serum OC level (r = 0.73, *p* < 0.001) and serum vitamin D concentration (r = 0.296, *p* = 0.01).

We have not found any significant correlation between duration of efavirenz therapy and serum level of vitamin D or bone biomarkers’ levels.

### Discussion

Considering high prevalence of vitamin D deficiency in HIV infected individuals, this population can benefits from vitamin D nutritional support. These patients usually have complex drug therapy regimen including antiretroviral drugs and prophylactic therapies for opportunistic infections. With respect to importance of treatment compliance in this group of patients, single dose supplementation may be preferred. This is the first interventional study that has evaluated the effects of single dose of vitamin D3 on the bone specific biomarkers in this population.

Bone specific markers are helpful biochemical indices to evaluate bone dynamic and metabolic status [7]. Effects of HIV infection and antiretroviral therapy on bone biomarkers in HIV positive individuals were reported [13-15]. Changes in the bone biomarkers reach to steady state approximately 3 months after exposure to conditions or drugs that affect the bone turn over metabolism [13,16]. We have measured the bone biomarkers at baseline and three months after vitamin D supplementation.

The patients’ median of vitamin D baseline serum levels were in moderate deficiency range. After three months of vitamin D supplementation, it elevated to normal level range that was far from toxic levels [17]. This intervention was effective, safe and convenient like other populations [18-20].

In this study, changes in the bone specific biomarkers’ concentrations clearly indicated that the single high dose of vitamin D3 can increase bone metabolic function and osteoblasts activity and bone formation were more enhanced. Osteoblast and osteoclast cells activity are correlate to each other via osteoprotegrin (OPG)/receptor activator of nuclear factor-kappa B (RANK)/RANK ligand (RANKL) system [21,22]. Recent studies confirmed existence of vitamin D receptor (VDR) on the osteoblasts and osteoclasts surface [23,24]. This vitamin can improve osteoblats activity, osteocalcin concentration and bone formation. Also it can increase bone resorption following increase in RANKL expression [25]. This remove and repair (remodeling process) is necessary for keeping skeletal strength and its architectures as well as repair the micro fractures and regulate calcium serum level [8].

HIV infected individuals’ bone health has been described in previous studies [2,3,9,14]. In vitro studies confirmed that HIV virus's antigens like gp120 and p55-gag can induce osteoblasts apoptosis and RANKL expression, resulting in increase bone resorption. Additionally antiretroviral drugs also can affect the bone [15]. Our data showed that osteocalcin as a bone formation biomarker is lower than desirable concentration in these patients and had positive correlation with vitamin D serum level. This finding is in agreement with the previous studies [14,26].

Following vitamin D supplementation in our patients, osteocalcin serum concentration increased about three fold compared with its baseline value but serum CTx concentration rose to a lesser extent. Increasing in concentration of bone formation marker followed by increasing in bone resorption marker level known as overall remodeling process activation that have positive balance on the bone formation. These results are in consistence with previous reports with triparetide, showed that activation of bone remodeling can result in new bone formation [27,28].

There are several limitations to our study. An important one is the lack of bone mineral density assessment in our patients. We did not detect any significant correlation between biochemical or bone markers with length of efavirenz therapy and it may be due to limited sample size of the study. Our study had not enough duration of follow-up to evaluate effect of vitamin D on bone fracture reduction.

### Conclusion

In conclusion our data suggests that in HIV-infected individuals under treatment with efavirenz, vitamin D deficiency is prevalent and consequently bone health may be affected. After supplementation with single dose of 300,000 IU vitamin D, the activation of osteoblasts and osteoclasts stimulates bone formation and resorption respectively with favorable bone formation without any adverse event.

### Method

This prospective interventional study was done in HIV Clinic of Iranian HIV/AIDS Research Center affiliated to Tehran University of Medical Sciences; the major referral center for HIV infected patients’ care in Tehran, Iran. HIV infected individuals aged 18 to 65 years old included in this study if they were under treatment with antiretroviral drugs including efavirenz 600 mg daily for at least one month. The Tehran University of Medical Sciences local ethical committee approved the study and all subjects entered in the assessment after giving written informed consent. We excluded individuals with known risk factors that could affect the bone turnover including history of hypogonadism, menopause, abnormal renal, hepatic or thyroid function tests, adrenal insufficiency, metabolic bone diseases, pregnancy and lactation, malignancy, immobility for more than one-week, and taking medications known to influence calcium or vitamin D metabolism or bone mass such as corticosteroids, thiazides, diuretics, anabolic steroids, and anticonvulsants. We also excluded subjects with a history of any vitamin D supplement intake during previous 3 months.

Demographic data of the patients were collected and following an overnight fasting, 10 ml venous blood sample were obtained and then serum was separated and froze at −70°C until time of analysis.

Patients’ serum levels of calcium (Ca), P, creatinine (Cr) and total ALP and albumin (Alb) were measured using standard automated equipment. Total serum concentration of Ca was corrected based on serum albumin according to; corrected total Ca (mg/ dl) = measured Ca (mg/dl) + 0.8 × [4 − Alb (g/dl)]. Patients’ GFR was calculated based on Cockroft-Gault formula. The serum PTH (Biomerica, Germany), OC (DIAsource, Belgium), CTx and vitamin D (25-OH vitamin D) (Immuno-diagnostic systems, United Kingdom) concentrations were measured using enzyme-linked immunosorbent assay kits.

For vitamin D deficient patients (serum vitamin D concentration less than 35 nmol/ml) 300,000 units vitamin D was administered intramuscularly. All included patients were followed for three months and after that all of the mentioned laboratory parameters were repeated at this time.

Data were analyzed using SPSS 16.0 software (SPSS, Chicago, IL, USA). Kolmogorov-Smirnov test was used to determine the normal frequency of different variables. Results were expressed as mean ± SD for parametric and median (range) for nonparametric data. Pearson and Spearman correlation coefficients were used to analyze the correlations, and paired t tests or Sign tests were used for comparing data before and after intervention. A p-value of less than 0.05 was considered statistically significant for all analyses.

## Competing interest

We have no received any fund for this research and all authors have not conflict of interest.

## Authors’ contributions

Hossein khalili: Designing of the study and coordinator, Maryam Etminani-Esfahani: Patients’ clinical assessment and data gathering, Sirous jafari: Patients’ selection and ordering of intervention, Alireza Abdollahi: Laboratory analysis, Simin Dashti-Khavidaki: Editing of the manuscript. All authors read and approved the final manuscript.
